# Psychometric Evaluation of the Persian Version of the Childbirth Experience Questionnaire (CEQ)

**DOI:** 10.1155/2020/6879283

**Published:** 2020-12-10

**Authors:** Samiyeh Kazemi, Anna Dencker, Farzaneh Pazandeh, Ali Montazeri, Sedigheh Sedigh-Mobarakabadi, Sepideh Hajian

**Affiliations:** ^1^Student Research Committee, Department of Midwifery and Reproductive Health, School of Nursing and Midwifery, Shahid Beheshti University of Medical Sciences, Tehran 19968-35119, Iran; ^2^Institute of Health and Care Sciences, Sahlgrenska Academy, University of Gothenburg, Gothenburg 457, 40530, Sweden; ^3^School of Health Sciences, University of Nottingham, Nottingham NG7 2RD, UK; ^4^Midwifery and Reproductive Health Research Center, School of Nursing and Midwifery, Shahid Beheshti University of Medical Sciences, Tehran 19968-35119, Iran; ^5^Population Health Research Group, Health Metrics Research Center, Iranian Institute for Health Sciences Research, ACECR, Tehran 13157-95795, Iran

## Abstract

Women's childbirth experience is an outcome indicator for evaluating maternity care. This study evaluated the psychometric properties of the Persian version of the Childbirth Experience Questionnaire (P-CEQ). The study recruited two hundred and fifty primiparous postpartum women in the 1-3 months following birth from one private and three public hospitals in Mazandaran province, Iran. First, face validity and content validity were evaluated. Moreover, confirmatory factor analysis (CFA) was conducted, and discriminant validity was assessed by applying the known-groups method. Intraclass correlation coefficient (ICC) was measured to confirm the stability and Cronbach's alpha to confirm the internal consistency. CFA also confirmed the values of fit indices (RMSEA = 0.05, SRMSR = 0.06, CFI >0.93, *χ*^2^/df = 1.80). ICC was 0.88 and Cronbach's alpha for all items was 0.85. Furthermore, discriminant validity of the P-CEQ was approved given that it effectively differentiated women whose stay in the labor unit exceeded twelve hours from those with a shorter stay. The P-CEQ questionnaire is a valid and reliable tool for assessing childbirth experiences. It is an easy-to-use questionnaire that can be used for evaluating quality of care in terms of women's childbirth experience. It can be used in maternity services that aim to improve quality of care during labor and childbirth.

## 1. Introduction

Childbirth is an invaluable process that affects the health of women and their babies and their emotional connection, as well as sexual activity and the desire to have further children [1, 2]. A positive childbirth experience creates a positive attitude toward normal childbirth by creating a sense of control, power, satisfaction, and reassurance for women [3, 4]. The negative childbirth experience is linked to lower rates of breastfeeding, increased maternal-infant communication disorders at birth, and may lead to postpartum depression and posttraumatic stress disorder (PTSD) [5, 6]. The World Health Organization (WHO) has issued guidelines recognizing a “positive childbirth experience” as a significant endpoint for all women undergoing labor [2].

In recent years, significant reductions in maternal and newborn mortality rates have been reported in Iran, mostly due to increased birth facilities and skilled birth attendants [7]. However, a review of the existing literature in Iran shows that the medicalization of childbirth has led to increasing medical interventions and technologies including early admission in labor, stimulation and induction of labor, episiotomy, and caesarean section [8, 9]. It is believed that the next priority in this country should be to improve the quality of childbirth care and women's childbirth experience and satisfaction [10].

There is consensus that women's childbirth experience has an important impact on quality assessment and improvement [2, 11]. The first step in improving childbirth care is to evaluate women's experience of childbirth. Therefore, there is a need for a valid and reliable tool. There are different tools for measuring the childbirth experience in different languages, but the Childbirth Experience Questionnaire (CEQ) [12] is a multidimensional tool that comprehensively evaluates women's perceptions of childbirth care. The CEQ validation was carried out in England, Spain, and more recently in China [13–15] and is widely used to evaluate the experiences of women in childbirth. The original Swedish version of the CEQ [12] was translated into Persian by Abbaspoor et al. [16]. However, the process of evaluating its psychometric properties was incomplete. Therefore, there was a need to evaluate the psychometric properties and to adapt it to the Iranian context. This study evaluated the CEQ's psychometric properties (validity and reliability) in Iran.

## 2. Materials and Methods

### 2.1. Study Setting

A cross-sectional study was conducted at one private and three public hospitals in Mazandaran province (North of Iran) from March to November 2019.

### 2.2. Sample Size Calculation

The sample size anticipated for the study was 220 women. This was based on a standard sample size which was ten times the number of items [17]. We also took missing items into consideration [18], and the final sample size was 250.

### 2.3. Sample and Procedure

The questionnaire was given to a convenience sample of women at discharge after childbirth. The eligible participant women comprised literate primiparous women aged over 18 years, undergoing vaginal birth and giving birth to a healthy baby without any complications at a gestational age at birth of 37-42 weeks after a singleton and full-term pregnancy. First, we contacted postpartum women at the time of discharge from the hospital and arranged the date of the interviews. The P-CEQ is a self-administered questionnaire and was completed by postpartum women 1-3 months after birth when they came to the health facilities to receive maternal and neonatal care. It took roughly 15-20 minutes to complete each questionnaire. Women's obstetric characteristics were collected using their medical records.

### 2.4. CEQ Questionnaire

The original CEQ was developed in Sweden [12]. It consists of 4 subscales namely: own capacity (8 items), professional support (5 items), perceived safety (6 items), and participation (3 items). The items were scored on a 4-point Likert scale: (1) Totally agree, (2) Mostly agree, (3) Mostly disagree, and (4) Totally disagree. The items regarding labor pain and senses of control and security were scored 0-100 on a visual analogue scale, which was converted to 1-4 as in the other items. The CEQ's scoring process has been detailed in literature [12].

### 2.5. Linguistic Validation

The original Swedish version [12] was translated into Persian by Abbaspoor et al. [16]. This Persian version was used with a few amendments to the items' wording. The interpretations of “labor and childbirth” were kept in all the items. However, in the earlier translated version, only the translation of “childbirth” was included. Moreover, the translation of “depressed” in item nine (I felt depressed) reflected sadness rather than depression. Item fourteen (My midwife devoted adequate time to my partner), which was excluded from the previous Persian translation, was also kept in the P-CEQ by replacing “my partner” with “my accompanying person.”

### 2.6. Evaluation of the Psychometric Properties

First, face validity and content validity were evaluated, and then, construct validity was assessed using confirmatory factor analysis and discriminant validity. The reliability was assessed using internal consistency and test-retest.

#### 2.6.1. Face and Content Validity

The face validity of this instrument was investigated both quantitatively and qualitatively. First, face validity was assessed by asking ten experts in midwifery and reproductive health to evaluate the questionnaire. Then, twenty nulliparous pregnant women attending health centers were asked to comment on the questionnaire in terms of appropriateness and relevance, ambiguity or possible misinterpretation of the phrases, and difficulty of the phrases and words. In the quantitative step, efforts were made to merge or eliminate similar phrases and to determine the importance of individual phrases as impact scores on the basis of a 5-point Likert scale. The items with an impact score exceeding 1.5 were found appropriate and kept for further analyses [19].

Additionally, content validity was confirmed on the basis of the experts' comments and quantified based on the Content Validity Ratio (CVR) and the Content Validity Index (CVI). CVRs of over 0.62, and CVIs of over 0.79 were considered valid [19].

#### 2.6.2. Construct Validity

Exploratory Factor Analysis (EFA) was not used to evaluate the questionnaire's factor structure, because we already had a model to test [12]. The Confirmatory Factor Analysis (CFA) was carried out instead. The fitness indices were used to assess the model's fitness. The fitness of the model was confirmed using the following thresholds: normed chi-square *χ*^2^/df <5.0, RMSEA <0.08, SRMSEA <0.08, and CFI ≥0.90 [20, 21].

#### 2.6.3. Discriminant Validity

The discriminant validity was evaluated by testing P-CEQ score differences between subgroups that were expected to differ in the key variables. The total score of the childbirth experience and its domains' scores were therefore compared among the study samples, which differed by type of birth, labor duration [12, 22], and oxytocin augmentation [23]. Other items such as hospital type and perineal status after birth were compared between the study samples. Based on previous research, it was hypothesized that women with shorter labor time and without oxytocin augmentation were expected to have a better childbirth. The effect size was calculated based on Cohen's description [24] and was computed as the difference between group mean scores divided by the pooled standard deviation of two groups. Values “between 0.2 and 0.5,” “between 0.5 and 0.8,” and “higher than 0.8” were considered low, moderate, and high, respectively [24]. The Mann–Whitney *U* test was used to contrast the hypotheses [20].

#### 2.6.4. Reliability

The reliability was determined using the internal consistency test (Cronbach's alpha coefficient) and test-retest reliability [25, 26]. A Cronbach's alpha of at least 0.70 was considered acceptable. The most acceptable test to determine stability is the intraclass correlation coefficient. Thus, test-retest reliability was measured using ICC, two-way mixed from a single measure [26]. To assess the reliability of the adapted version of the questionnaire in terms of stability, a subsample of 25 eligible women completed the questionnaire twice at a two-week interval. This formula was used for calculating ICC: MSR − MSE/MSR + (*k* − 1)MSE + (*k*/*n*) (MSC − MSE) [26], and ICC of 0.6 or above was acceptable.

### 2.7. Statistical Analysis

Data were analyzed using SPSS Statistics for Windows version 20.0 (SPSS Inc., Chicago, IL, USA), Mplus7.4 and R X64 3.5.1 (psych package), and LISREL 8.8). The construct validity was assessed by confirmatory factor analysis. The Mann–Whitney *U* test was used to contrast the hypotheses of discriminant validity [17, 20, 21].

### 2.8. Ethical Considerations

This study was approved by the Ethics Committee of Shahid Beheshti University of Medical Sciences, Tehran, Iran (Code: IR.SBMU.PHARMACY.REC.1397.028). First, permission was sought from the study hospitals. The research objectives were explained to the participants, and they were also assured of their right to withdraw from the study at any stage of the study. Those who agreed and signed the consent form participated in the study.

## 3. Results

### 3.1. Description of Sample

Out of 347 eligible participants, 250 (72%) women with a mean age of 30 ± 5.8 years consented to participate in the study. Labor lasted more than twelve hours in 27% of the participants, and 75% underwent episiotomy.  Table 1 presents the demographic information of the participant women.

### 3.2. Descriptive Statistics of the P-CEQ

The mean score of the overall childbirth experience was 59.08 ± 14.46. The mean scores of “own capacity,” “professional support,” “perceived safety,” and “participation” were 22.79 ± 5, 13.77 ± 3.77, 13.91 ± 3.73, and 8.61 ± 3.07, respectively.

### 3.3. Psychometric Properties

#### 3.3.1. Face and Content Validity

Some of the items were slightly modified based on the experts' comments during the face validity evaluation. Moreover, twenty nulliparous pregnant women identified all the items of the questionnaire to be transparent and easy to comprehend. The impact score ranged from 3.0 to 4.0 for each item (Table 2). According to the comments of ten experts, the impact scores of the items were calculated. The estimated CVI and CVR values were in the ranges of 0.83–1.00 and 0.80–1.00, respectively. All the items were therefore kept in the questionnaire (Table 2).

#### 3.3.2. Construct Validity

**Figure 1:**
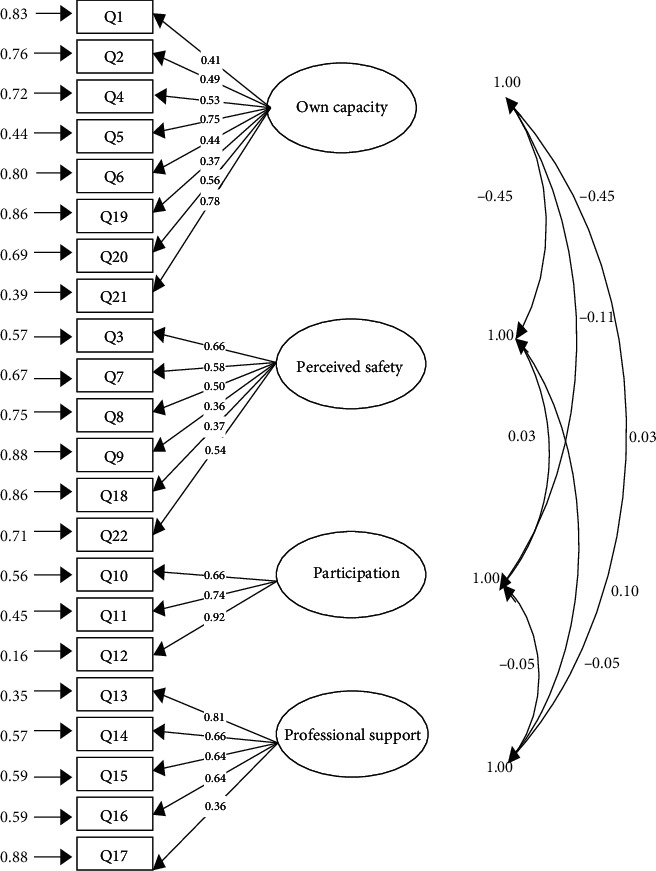
The Persian model of the CEQ.

The fitness of the CFA was required for the evaluation of the factor structures. Acceptable values of fit indices indicated a good model fit (RMSEA = 0.05, SRMSR = 0.06, GFI = 0.91, CFI >0.93, *χ*^2^/df = 1.80) (Table 3).  Figure 1 illustrates the four-factor model of the P-CEQ. The minimum and maximum coefficients of the item-scale relationship were 0.36-0.92. Moreover, all coefficients of the item-scale relationship in the CFA were significant (*P* < 0.001), indicating that all items were significantly correlated with their factors.

#### 3.3.3. Discriminant Validity

Discriminant validity was evaluated using the known-groups method and the independent Mann–Whitney *U* test to investigate the intergroup difference in the overall scores of childbirth experience and its domains. The total P-CEQ score and the scores of “own capacity” and “perceived safety” were significantly higher in the women with a shorter stay in the labor unit (less than 12 hours) than in those with a longer stay, suggesting small effect sizes. According to Table 4, no significant differences were observed in the total P-CEQ score and the scores of its domains between the women with or without oxytocin augmentation during labor (*P* = 0.91), hospital type (*P* = 0.92), type of birth (*P* = 0.030), and the state of the perineum following birth (*P* = 0.07).

#### 3.3.4. Reliability Test

Cronbach's alpha was estimated at 0.85 for the whole P-CEQ, 0.84 for “own capacity,” 0.69 for “participation,” 0.92 for “professional support,” and 0.78 for “perceived safety.” The overall ICC of the P-CEQ was 0.88, indicating that reliability for test-retesting was excellent, as shown in Table 5.

## 4. Discussion

This study evaluated the psychometric properties of the CEQ and adapted it to the Iranian culture with a similar internal consistency to its English version [13]. The P-CEQ was found to be a valid and reliable measure of childbirth experience among Iranian women. The total Cronbach's alpha calculated for the P-CEQ was consistent with that obtained for its Spanish (0.88) and Chinese versions (0.88) compared to the Cronbach's alpha obtained for the English version (0.90), although it was not reported for the Swedish version [12–15]. The fit indices were verified by the CFA performed in the present analysis, and items 9, 17, and 18 of the P-CEQ were weakly correlated with the given factors. This may indicate that the data conveyed by items 17 and 18 were also included in items 13 and 16. The insignificant correlation of item 18 can be explained by the participants' inadequate understanding of this item and that of item 9 by its incompatibility with the setting and facilities of childbirth units in Iran.

Our study reported higher scores in those with a shorter stay in the labor unit, though the effect size was very small. However, the Swedish study [12] reported significantly higher scores for the domains of the CEQ in women with a longer labor and in those without oxytocin augmentation. The discrepancy in results can be related to differences in the childbirth context and the samples between Sweden and Iran. For instance, spontaneous onset of labor was reported in the majority of the Swedish women, whereas merely 12% underwent instrumental birth. The largest effect size was obtained through comparing spontaneous vaginal birth with instrumental childbirth (instrumental vaginal birth and cesarean section) in the study conducted in Sweden [12]. The present study did not address this factor given that it did not include women undergoing cesarean section and only seven out of the 250 participants in our study underwent instrumental vaginal birth.

The P-CEQ is a tool for evaluating women's childbirth care experiences. Given that women are increasingly vulnerable to the onset or relapse of mental disorders during pregnancy and the postpartum period, this screening tool may help providers to identify postpartum women with negative health experiences who are at risk of PTSD and serious mental disorders [27]. This may promote the long-term wellbeing of women and their babies [28] by supporting women at higher risk and referring them for nonpharmacological and psychopharmacological treatments [29].

## 5. Limitations

This study was carried out in both public and private hospitals that could be the strength of this study. Some clinical data such as the duration of labor (shorter or longer than 12 hours), oxytocin augmentation (use or no use), and the precise timing for the onset of labor were obtained using women's medical records, which can be considered a limitation of this study. The participants of our study were primiparous women with low-risk pregnancies. Multiparous women and those with complicated pregnancies may have different experiences. Thus, the use of the P-CEQ in multiparous women and those with high-risk pregnancies is warranted.

## 6. Conclusions

The P-CEQ is a valid and reliable questionnaire for evaluating women's experiences during labor and childbirth and can be used in settings that aim to improve the quality of care during labor and childbirth.

## Figures and Tables

**Table 1:** Demographic and obstetric characteristics of the postpartum women.

Demographic and obstetric characteristics	*n* (250)	(%)
Education		
High school or lower	103	41.2
College or higher	147	58.8
Occupation		
Employed	209	83.5
Home-maker	23	9.2
Self-employed	18	7.2
Abortion history		
Yes	48	19.2
No	202	80.8
Oxytocin augmentation		
Yes	169	67.6
No	81	32.4
Labor duration longer than 12 hours		
Yes	67	26.8
No	183	73.2
Type of childbirth		
Spontaneous vaginal	243	97.2
Instrumental	7	2.8
Perineal status after birth		
Tears (grade 1 and 2)	63	25.2
Episiotomy	187	74.8
Hospital type		
Public	211	84.4
Private	39	15.6
	Mean	SD
Maternal age (years)	26	3
Gestational age (weeks)	38.5	1.3

**Table 2:** The impact score, CVI, and CVR for the P-CEQ.

Items	Impact score (*n* = 20 mothers)	CVI (*n* = 10 experts)	CVR (*n* = 10 experts)
Item 1	3.6	0.98	1
Item 2	3.2	0.83	0.80
Item 3	3.8	1	1
Item 4	3.7	1	1
Item 5	3.2	0.83	0.80
Item 6	4	0.96	1
Item 7	3.3	0.83	0.80
Item 8	4	1	1
Item 9	3	0.90	1
Item 10	3.5	0.90	1
Item 11	4	1	1
Item 12	3.2	0.83	0.80
Item 13	4	1	1
Item 14	3	0.90	1
Item 15	3.3	0.83	0.80
Item 16	4	0.96	1
Item 17	3.2	0.83	0.80
Item 18	3	0.90	1
Item 19	3.5	0.90	1
Item 20	3.7	1	1
Item 21	4	1	1
Item 22	4	0.96	1

**Table 3:** Fit indices of the four-factor model of the P-CEQ.

Model	AGFI	GFI	SRMSR	RMSEA	NFI	CFI	*X* ^2^
P-CEQ (22 items)	0.90	0.91	0.06	0.05	0.92	0.93	263.07

AGFI: Adjusted Goodness of Fit Index; GFI: Goodness of Fit Index; SRMSR: Standardized Root Mean Square Residual; RMSEA: Root Mean Square Error of Approximation; NFI: Normed Fit Index; CFI: Comparative Fit Index; *X*^2^: Satorra-Bentler *χ*^2^.

**Table 4:** P-CEQ overall and subscale scores by different groups (*n* = 250).

Groups	Own capacity	Participation	Professional support	Perceived safety	Mean CEQ score
Hospital type					
Public (*n* = 211)	2.3 (0.6)	2.6 (0.6)	2.8 (0.6)	2.7 (0.5)	2.4 (0.5)
Private (*n* = 39)	2.3 (0.5)	2.5 (0.5)	2.5 (0.5)	2.2 (0.4)	2.3 (0.4)
*P* value	0.80	0.40	0.080	0.99	0.92
Cohen's effect size	0.05	0.10	0.09	0.33	0.22
Labor duration					
≤12 h (*n* = 193)	2.5 (0.6)	2.6 (0.7)	2.7 (0.7)	2.6 (0.7)	2.6 (0.5)
>12 h (*n* = 67)	2.3 (0.7)	2.5 (0.8)	2.5 (0.8)	2.3 (0.8)	2.3 (0.6)
*P* value	0.001	0.510	0.087	0.005	0.007
Cohen's effect size	0.33	0.10	0.15	0.28	0.26
Oxytocin augmentation					
Yes (*n* = 169)	2.4 (0.7)	2.5 (0.7)	2.7 (0.7)	2.5 (0.7)	2.5 (0.6)
No (*n* = 81)	2.4 (0.6)	2.6 (0.6)	2.6 (0.7)	2.5 (0.7)	2.5 (0.5)
*P* value	0.852	0.429	0.081	0.998	0.912
Cohen's effect size	0.06	0.11	0.07	0.00	0.02
Type of birth					
Spontaneous vaginal (*n* = 243)	2.8 (0.5)	2.8 (0.8)	3.5 (0.5)	3 (0.6)	3 (0.4)
Instrumental (*n* = 7)	2.5 (0.5)	2.6 (0.8)	3.5 (0.4)	2.6 (0.6)	2.9 (0.4)
*P* value	0.082	0.18	0.50	0.01	0.030
Cohen's effect size	0.30	0.20	0.01	0.30	0.30
Perineal status after birth					
Episiotomy (*n* = 187)	2.7 (0.6)	2.8 (0.9)	3.6 (0.6)	3 (0.7)	3.1(0.6)
Tears (grade 1 and 2) (*n* = 63)	2.6 (0.5)	2.6 (0.8)	3.5 (0.5)	2.8 (0.6)	2.9 (0.4)
*P* value	0.42	0.50	0.43	0.035	0.07
Cohen's effect size	0.36	0.30	0.04	0.22	0.36

Data presented as mean (SD); Mann–Whitney *U* test was used to compute *P* values. The overall score of the CEQ was the mean score of the four subscales.

**Table 5:** The reliability of the overall CEQ and subscales.

Overall CEQ and subscales	Number of items	Cronbach's alpha	ICC
Own capacity	8	0.84	0.84
Professional support	5	0.92	0.89
Perceived safety	6	0.78	0.87
Participation	3	0.69	0.85
Overall	22	0.85	0.88

CEQ: Childbirth Experience Questionnaire; ICC: intraclass correlation.

## Data Availability

The data will be available on reasonable request from the corresponding author (FP).
